# Patient preferences for allogeneic haematopoietic stem cell transplantation: how much benefit is worthwhile from the patient’s perspective?

**DOI:** 10.1007/s00520-020-05816-z

**Published:** 2020-10-17

**Authors:** Nicolas Leuthold, Marco Cattaneo, Jörg Halter, Claudia Hügli, Monika Kirsch, Anna Petropoulou, Tobias E. Erlanger, Sabine Gerull, Jakob Passweg, Alix O’Meara Stern

**Affiliations:** 1 University of Basel, Basel, Switzerland; 2 Department of Clinical Research, University of Basel and University Hospital Basel, Basel, Switzerland; 3 Division of Haematology, University Hospital Basel, Basel, Switzerland; 4 Department of Pediatric Oncology, Haematology and Immunology, University of Heidelberg, Heidelberg, Germany; 5 Department of Anaesthesia, University Hospital Basel, Basel, Switzerland; 6 Department of Oncology, Cantonal Hospital Neuchâtel, Neuchâtel, Switzerland

**Keywords:** Patient preferences, Decision-making, Allogeneic stem cell transplantation, Graft-versus-host disease

## Abstract

Oncological studies have shown that patients consider small benefits sufficient to make adjuvant chemotherapy worthwhile. We sought to determine the minimal survival benefits that patients considered enough to legitimate allogeneic haematopoietic stem cell transplantation (HCT) and the factors associated with patient preferences. One hundred eighty-four patients having previously received allogeneic HCT at our centre were included and completed a questionnaire exploring patient expectations elicited by time trade-off scenarios as well as quality of life (QoL), symptoms of graft-versus host disease (GvHD) and sociodemographic characteristics. The majority of patients considered a minimal survival benefit of at least 5 (38.6%) or 10 years (41.9%) sufficient to justify HCT, with less than 5% considering survival < 1 year sufficient to warrant HCT. In terms of minimal cure rate, a cumulative 14.8% of patients accepted cure rates below 30% and 30.6% rates below 50%. Likelihood-ratio tests were significant for the effect of age at transplant on expected minimal survival (*p* = 0.007) and cure rates (*p* = 0.0001); that is, younger patients at HCT were more likely to accept smaller survival and cure rates. Pre-transplant risk score, QoL, GvHD score and sociological factors did not seem to influence patients’ expectations. In conclusion, patient expectations of treatment were much higher than what had been reported in oncological studies. Patients who experienced HCT considered a survival superior to 1 year and cure rates above 50% sufficient to make it worthwhile. Younger patients were more likely to accept smaller benefits; no other predictors for preferences could be detected.

## Introduction

Allogeneic haematopoietic stem cell transplantation (HCT) is a procedure that aims at curing a wide array of diseases ranging from myeloid and lymphoid neoplasia to non-malignant disorders. Cure rates of up to 70% can be achieved depending on the underlying disease, patient, donor and transplant characteristics [1]. At the same time, allogeneic HCT can elicit side effects, infection and graft-versus-host disease (GvHD) being the most determinant factors for the patient’s outcome. Serious adverse effects such as uncontrollable opportunistic infections or severe steroid-refractory GvHD can lead to death with documented treatment-related mortality rates of up to 50% [2]. Late effects such as cardiovascular, secondary neoplastic and infectious complications represent unique challenges in the care of long-term survivors [3].

While some side effects such as mucositis will pass, others may become chronic. Chronic GvHD has a strong impact on the patient’s course and quality of life [4, 5]. From the patients’ point of view, chronic GvHD can be perceived as an exchange of diseases. As such, the indication for allogeneic HCT must be considered carefully and the possible benefits and risks discussed in detail. Practice guidelines, risk-stratification scoring systems, tumour board discussion, and long-standing experience support stem cell transplantation units in such decisions [6, 7]. For the patient as an individual, however, the impact of transplantation and its true outcome remain difficult to foresee.

In solid tumour oncology, many trials have been conducted in order to assess patient preferences and their perception of the possible benefits and risks of adjuvant chemotherapy for breast, lung and colon cancer [8–11]. As an example, an Australian study based on the “time trade-off” interview model, i.e. envisioning possible hypothetical life-prolonging scenarios through chemotherapy of breast cancer, revealed unexpected results. Almost half of the patients declared that in their opinion, a relative increase of 1% of their chances of survival over the next 5 years would justify undergoing the 6-month chemotherapy regimen they had previously received [12].

Our scientific knowledge of the patient’s decision-making benchmarks, perceptions and expectations in the setting of allogeneic HCT is scarce. In this specific context of high cure rates associated with considerable risks of severe and potentially non-reversible adverse events, additional information would be useful. The aim of this survey was to gain better scientific insights into the expectations of allogeneic HCT from the patient’s point of view and to contribute to providing due diligence in pretransplant counselling.

## Patients and methods

The local ethical committee approved this single-centre cross-sectional observational questionnaire-based study. Patients having received allogeneic HCT prior to October 2018 at the University Hospital of Basel and identified as fulfilling inclusion criteria (minimum age of 18 years) were approached for informed consent. Our centre serves multiple language regions in Switzerland (German, Italian and French speaking); patients not fluent in German the project language were excluded. One hundred eighty-four patients in the full analysis set (FAS) provided written consent and completed the questionnaire in time. According to protocol pre-specified questionnaire quality assurance rules, two questionnaires yielding spurious answers to the control questions and one questionnaire delivering a non-completion rate > 15% were not included in the per protocol set (PPS).

### Written questionnaire

The survey consisted of 11 pages of written questionnaire including sociodemographic questions, a quality of life (QoL) score [13], a distress thermometer [14], and a GvHD scoring tool corresponding to a modified PROVIVO questionnaire [15, 16]. The questionnaire was submitted for revision to HCT specialists and patient delegates before its implementation.

### Time trade-off questions

Patients were presented with two control questions and a question addressing their current health state. Patients further chose from hypothetical minimal survival benefits and minimal cure rates allogeneic HCT would have to provide in order to be deemed worthwhile. Health benefit questions were formulated on the premise of “based on your experience, what period of survival deriving from HCT would make it worthwhile?”. Finally, patients were asked whether they considered having made the right choice by undergoing allogeneic HCT or whether they would have chosen differently if they had had another chance to decide.

### Quality of life score

The patients’ current health state was addressed in the form of a modified RAND 36-Item Short Form Survey and analysed by standard methods according to Ware & Sherbourne [13]. The QoL score was calculated by adapting the RAND 36-Item Health Survey 1.0 scoring method as follows: the answers to each one of the 36 single questions were scored on a 0 to 100 range, so that a high score defines a more favourable health state; the lowest and highest possible scores were 0 and 100, respectively; and the possible scores were equally spaced on the 0 to 100 range. These 36 single scores were averaged to obtain the following nine-scale scores: physical functioning, role limitations due to physical health, role limitations due to emotional problems, energy/fatigue, emotional well-being, social functioning, pain, general health and health change. The resulting nine-scale scores were finally averaged to obtain the QoL score, which is thus a weighted average of the 36 single scores.

### GvHD score

GvHD score was assessed in a short version of the PROVIVO symptom experience questionnaire [15, 16]. The calculation of the GvHD score was built in analogy to the QoL score. The answers to each one of the 42 single questions were scored on a 0 to 100 range, a high score defining a higher burden of GvHD symptoms; the lowest and highest possible scores were 0 and 100, respectively; and the possible scores were equally spaced on the 0 to 100 range. These 42 single scores were averaged to obtain the following five-scale scores: physical symptoms, emotional and cognitive symptoms, sexuality, effects on daily life and the frequency of infections. The resulting five-scale scores were finally averaged to obtain the GvHD score, which is thus a weighted average of the 42 single scores.

### Statistics

All analyses were conducted using the statistical software package R [17], using “two-sided” statistical tests and confidence intervals with standard significance and confidence levels *α* = 5% and (100% − *α*) = 95%, respectively. Missing data was handled by pairwise deletion (available-case analyses). The FAS consisted of all 184 patients who provided written consent and returned the questionnaire in time. The PPS consisted of 181 patients as detailed above. All statistical analyses were performed on the FAS, except for sensitivity analyses performed on the PPS.

### Endpoints

The primary endpoint was the minimal health benefits that patients judged sufficient to make allogeneic HCT worthwhile, with a particular focus on the minimal survival and cure rates. The distributions of the primary endpoints are described numerically and graphically using cumulative proportions and the corresponding confidence intervals.

Possible patient and disease characteristics affecting the primary endpoints were analysed by multivariable ordinal logistic regressions. The results were compared with those of multivariable linear regressions. The following covariates were included in the multivariable regression models: QoL score, GvHD score, pre-transplant risk score, age at transplant, time between transplant and survey, family donor and previous transplant. Finally, the effects on the primary endpoints of grouping by sociological factors were tested by Kruskal–Wallis’ one-way analysis of variance.

## Results

Seventy-four female and 110 male patients having previously received allogeneic HCT between 1980 and 2018 at our centre were included. Numerical participation according to decade of allogeneic HCT was crescent (1980–1989: 7; 1990–1999: 10; 2000–2009: 46; 2010–2019: 121). The median age at the time of transplant was 50 years (IQR: 39–60 years). Forty-eight patients had received more than one HCT, primarily autologous with ten patients having received previous allogeneic HCT. Haematopoietic stem cell donors at last HCT were either unrelated (50%), HLA-identical siblings (43.5%), mismatched related (3.3%), syngeneic (1.1%) or haploidentical (2.2%). The median time from transplant to survey was 6.64 years (IQR: 3.28–12.06). Patient and transplant characteristics are presented in Table 1. The data was complete, except for disease status at allogeneic HCT in 0.5% and on the occurrence of relapse or progression after allogeneic HCT in 1.1% of cases.

**Table 1:** Patient and transplant characteristics

Variables	*N*/median (%/IQR)
*N*	184
Median age, years	50 (39–60)
Male/female	110/74
Disease	
Acute lymphoblastic leukaemia	31 (16.8%)
Acute myelogenous leukaemia	49 (26.6%)
Bone marrow failure	8 (4.3%)
Myelodysplastic syndromes	20 (10.9%)
Myeloproliferative neoplasms	36 (19.6%)
Lymphoma	22 (12%)
Plasma cell disorders	9 (4.9%)
Chronic lymphocytic leukaemia	9 (4.9%)
Donor	
HLA-identical sibling	80 (43.5%)
HLA-matched unrelated	92 (50%)
Mismatched related	6 (3.3%)
Syngeneic	2 (1.1%)
Haploidentical	4 (2.2%)
Median pretransplant EBMT risk score	4 (2–5)
Disease status at transplant	
Progressive disease	41 (22.4%)
Stable disease	27 (14.2%)
Partial remission	17 (9.3%)
Complete remission	99 (54.1%)
Stem cell source	
Bone marrow	29 (15.8%)
Peripheral blood	154 (83.7%)
Cord blood	1 (0.5%)
Previous HSCT	48 (26.1%)
Social circumstances, marital status	
Single/divorced	25/6 (13.7%/ 3.3%)
Married/ has a life partner	111/27 (60.7%/ 14.8%)
Has dependents (children +/or adults)	14 (7.7%)
Was a previous cancer care giver	107 (58.8%)
Highest earned degree of education	
Compulsory education	15 (8.2%)
Apprenticeship	102 (56%)
Secondary education	12 (6.6%)
Tertiary education	53 (29.1%)
Religious affiliation	
Yes	22 (12.4%)
No	119 (66.9%)
No religious beliefs	37 (20.8%)

Categorical variables are listed as frequencies and percentages and numerical variables as median and interquartile range

### Primary analysis

**Fig. 1:**
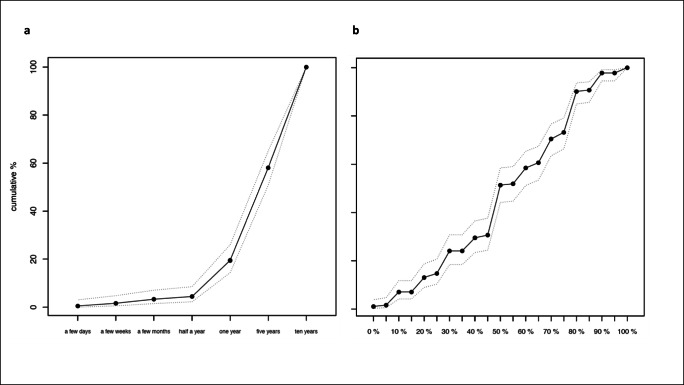
Minimal health benefit expectations. Panel a: cumulative proportions and corresponding confidence intervals of patients according to minimal survival considered sufficient to warrant undergoing allogeneic HCT. Panel b: cumulative proportions and corresponding confidence intervals of patients according to minimal cure rated considered sufficient to warrant undergoing allogeneic HCT

The minimal health benefits patients judged sufficient to make HCT worthwhile differed greatly from what had previously been reported in solid tumour oncology [11]. The majority of patients considered a minimal survival benefit of at least 5 (38.6%) or 10 years (41.9%) to legitimate allogeneic HCT. Less than 5% of all patients considered a survival benefit of less than 1 year sufficient to warrant undergoing HCT (Fig. 1, panel a). A minimal cure rate of 50% constituted an important threshold in our patient population preferences when evaluating minimal health benefits, with only a cumulative 14.8% of patients accepting cure rates below 30% (Fig. 1, panel b). Sensitivity analyses on the PPS showed only minimal differences.

### Secondary endpoints

The majority of patients reported experiencing little distress at the time of the survey: 67.5% responded with a score ≤ 3, the highest level of distress reported being 9 in 3% of patients. Median (IQR) of total QoL score was 75.5 (58.4–86.2) indicating good general health-related QoL. When broken down in physical and mental health sections, the median physical health QoL composite was 75.25 (IQR: 55.29–84.67) which did not vary greatly from the median mental health QoL composite of 78.54 (IQR: 65.75–88.42). Interestingly, 94% of all patients viewed undergoing allogeneic HCT as the right decision in their individual case. Only 1.6% admitted they would have opted against HCT in retrospect. The remaining 4.4% remained inconclusive about their choice of undergoing HCT.

GvHD scoring reached a median of 17.03 (IQR: 10.63–29.63), indicative of a low disease burden from GvHD symptoms. Patients reporting low QoL or a high GvHD score were more likely to answer they would not opt for HCT if they had another chance to decide (univariable ordinal logistic regressions: *p* < 0.001 for both QoL and GvHD scores). There was a strong correlation between low QoL and more severe GvHD scoring (*p* value < 0.001).

**Fig. 2:**
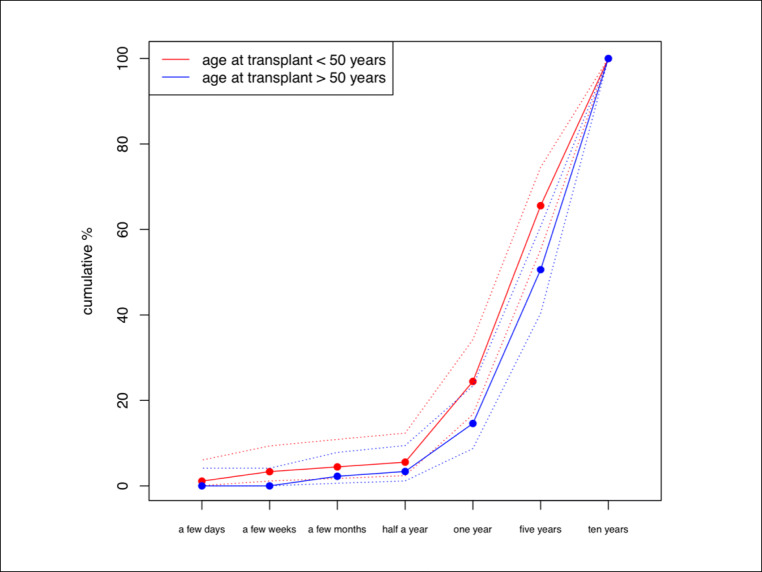
Minimal survival expectations according to age at allogeneic HCT

The single statistically significant predictor of acceptance of smaller benefits was the patients’ age at transplant, as seen in multivariable ordinal logistic regression models (Table 2). Likelihood-ratio tests were significant for the effect of age at transplant on expected minimal survival (*p* = 0.007) and minimal cure rates (*p* = 0.000). Patients aged under 50 years at transplant required smaller benefits from allogeneic HCT to perceive treatment worthwhile (Fig. 2). The multivariable linear regression models (Table 3) confirmed these results. The odds of higher survival expectations increased by 3.5% (1.035; 95% CI: 1.009–1.063) for each additional year of age at transplant when all other covariates such as previous HCT, family donor, time between transplant and survey remained unchanged.

**Table 2:** Likelihood-ratio tests for the effect of each covariate on expected minimal survival and minimal cure rates according to the multivariable ordinal logistic regression (FAS)

	Minimal survival *p* value	Minimal rate of cure *p* value
QoL score	0.598	0.476
GvHD score	0.636	0.549
Pre-transplant risk score	0.134	0.089
Age at transplant	0.007	0.000
Time between transplant and survey	0.589	0.057
Family donor	0.509	0.813
Previous transplant	0.676	0.882

**Table 3:** Multivariable linear regression of covariates influencing minimal survival and minimal cure rate expectancies (FAS)

	Minimal survival *p* value	Minimal rate of cure *p* value
QoL score	0.955	0.754
GvHD score	0.398	0.371
Pre-transplant risk score	0.194	0.092
Age at transplant	0.022	0.000
Time between transplant and survey	0.554	0.080
Family donor	0.315	0.824
Previous transplant	0.789	0.776

There was a trend to expect higher benefits with respect to minimal cure rates (*p* = 0.08) when the time between transplant and survey dated back longer in the multivariable linear regression model. No statistically significant effect on patient preferences could be shown for the prespecified factors: QoL score, GvHD score or pre-transplant risk score. No association was observed between patient preferences and donor-patient kinship or if the patient had previously received HCT as detailed in Table 3.

Sociological factors such as highest level of education, close family structures or religious affiliation did not seem to impact on patients’ expectations. No statistically significant correlation between minimal survival advantage or minimal cure rates could be detected in relation to marital status (*p* = 0.09, resp. *p* = 0.32), previous experience as a cancer caregiver (*p* = 0.12, resp. *p* = 0.89) or highest degree of education (*p* = 0.78, resp. *p* = 0.07). A trend to consider smaller cure rates worthwhile (*p* = 0.05) with no correlation to a minimal survival advantage (*p* = 0.45) was perceived in patients declaring religious affiliation.

## Discussion

Most of the patients judged a survival benefit superior to 1 year to be sufficient to legitimate allogeneic HCT, while less than 5% considered survival below the 1-year threshold to be worthwhile. These results differ clearly from the results on patients’ perspectives in solid tumour oncology where even trivial benefits of 0.1% supplementary cure rates or survival advantages of 1 day justified treatment in the patient’s opinion [11, 18, 19]. Patients who underwent allogeneic HCT expected a long survival and high cure rates, possibly reflecting awareness of the higher stakes, complexity and interpersonal dependency of this type of treatment.

In contrast to similar studies in solid oncology, no consistent correlation could be found between the patients’ health condition, social circumstances, educational status or belief and their expectations. The only covariate having a statistically significant impact on the level of the patients’ perspectives was the age at transplant. Not surprisingly, younger patients were more willing to accept smaller benefits in term of survival or cure rates than older patients. Other factors, such as pre-transplant risk score or consequences of the treatment, in particular a high symptom burden, did not influence significantly on the patients’ expectations. Neither did the patient’s own perception of his health condition, as illustrated by the modified QoL score, seem to affect patient preferences. Our initial hypothesis that long-term complications of allogeneic HCT such as chronic GvHD or loss of QoL would affect patients’ stance on the treatment could not be confirmed by this trial, possibly an effect of higher sampling probability for survivors with a favourable outcome and low GvHD symptom burden. Low QoL or a high GvHD score did however make the expression of regret of having undergone allogeneic HCT more probable, underlining the importance of our commitment to continuous care of this group of patients.

There are many possible explanations for the differences in outcome observed between our study and similar surveys in solid tumour oncology as well as limitations to our results. Due to the relatively small sample size, checking the assumptions tested is difficult. Moreover, the study was single centred and its generalizability may be debated. An important bias to our results originates in our patient collective which, inherent to the nature of the study, withholds a selection bias for a group of patients with more favourable allogeneic HCT outcome. While all patients at any timepoint after matched and unmatched allogeneic HCT were invited to participate, the study population was mainly composed of patients with a longer follow-up, almost exclusively after matched transplantation, reporting little distress and good QoL. Patients in a better health state and having benefited from a better outcome were more likely to participate in the study and possibly represent a certain opinion more strongly. Obviously, patients dying early after HCT were not part of the study.

Another possible bias stems from the questionnaire-based design of the study. The questionnaire contained non-trivial written questions that could have been misunderstood; additionally respondent survey fatigue could have occurred [20]. Prespecified questionnaire quality assurance rules were set to mitigate these risks. A personal interview method would have lessened the risk of misunderstanding and helped maintain respondent survey motivation, while the written questionnaire carried the asset of minimizing the influence an interviewer could take on the patient during personal interaction. Furthermore, a previous trial conducted in the setting of adjuvant chemotherapy for colon cancer compared personal interview and questionnaire-based systems, indicating only insignificant differences in validity and feasibility between the two methods [11].

The retrospective nature of the questionnaire diminishes comparability of the results. Patients are inherently biased by their own experience influencing their hypothetical expectations in allogeneic HCT, the strength of this particular study collective being that only patients with experience of allogeneic HCT were interrogated. Limitations due to recall bias are enhanced by a long interval between the event and the time of questioning as reflected by the long median time from transplant to survey (6.64 years). Our understanding would be more complete if information on patient expectations were collected and compared at different points in a prospective manner along the timeline of transplantation (e.g. before HCT, at day 30, 100, 1 year, and 5 years) and would enrich the cohort with the perspectives of patients experiencing detrimental complications of HCT. We do not know how patients responding to the questionnaire after HCT would have responded prior to HCT, had they had the opportunity. Patients many years after HCT may consider HCT only worthwhile if providing at least the survival benefit already experienced. However, we found no correlation between time interval from HCT and expected benefit.

In this study, we sought to bring more insight into patients’ expectations and to provide a framework of factors that might influence their preferences in the context of allogeneic HCT. While clinicians must provide evidence-based recommendations and inform in detail on the pros and cons of allogeneic HCT, room should be left for personal nuances and the individual dimensions of the decision-making process. The practical questions we attempted to answer in this trial were what benefit is sufficient for allogeneic HCT to be offered from the patient’s point of view, and are there differences in the minimal expectations depending on the patient’s context.

Unlike previous experience in solid tumour oncology, expected minimal health benefits in term of survival and cure rates after allogeneic HCT were non-trivial and the single statistically significant predictor of acceptance of smaller benefits was a younger age at transplant. In conclusion, most patients in this cohort, almost exclusively made up of patients after matched allogeneic HCT, considered a survival superior to 1 year and cure rates above 50% sufficient to make it worthwhile. Younger patients more willingly accepted smaller benefits, while no other predictors for preferences could be elicited in our trial. Obtaining individual patient preferences for allogeneic HCT was feasible, and additional studies would be of value in providing further guidance on tailoring our recommendations to patients’ needs.

## Data Availability

The data that support the findings of this study are available from the corresponding author, Alix O’Meara Stern, upon reasonable request.
